# Use of contrast-enhanced post-mortem computed tomography to diagnose intestinal perforation caused by malpractice: A case report

**DOI:** 10.1097/MD.0000000000015042

**Published:** 2019-04-05

**Authors:** Zhuoqun Wang, Kaijun Ma, Lei Wan, Donghua Zou, Ningguo Liu, Yijiu Chen

**Affiliations:** a Shanghai Key Laboratory of Forensic Medicine, Shanghai Forensic Service Platform, Academy of Forensic Science, Shanghai; b Shanghai Key Laboratory of Crime Scene Evidence, China.

**Keywords:** intestinal perforation, medical dispute, post-mortem computed tomography, post-mortem computed tomography angiography, virtopsy

## Abstract

**Rationale:**

The application of post-mortem computed tomography (PMCT) and PMCT angiography (PMCTA) is becoming increasingly common in forensic investigations. One of the most frequently applied techniques today is PMCTA. However, few studies have focused on the application of contrast-enhanced PMCT of hollow organs such as the gastrointestinal tract. The intestine is a special digestive organ with a complicated anatomical structure; it is difficult to separate in medicolegal investigations, during which new rupture may occur, affecting the examiner's judgment. Moreover, the formalin-fixed intestine is more difficult to separate because of its increased brittleness. In the present case, the authors applied contrast-enhanced PMCT to the diagnosis of intestinal perforation caused by a medical accident.

**Patient concerns:**

A 67-year-old woman with cholecystitis underwent laparoscopic cholecystectomy in the hospital. The gallbladder was successfully removed, but the doctor was suspected to have accidentally perforated her intestinal tract with the laparoscopic machinery. The patient developed severe peritonitis and died after the operation.

**Diagnosis, interventions, and outcomes:**

Contrast-enhanced PMCT with isolation of the intestinal tract was performed after dissection of the body. The results suggested that the contrast agent flowed out through the rupture. The autopsy and histological examination revealed a perforated crevasse, confirming the cause of peritonitis while excluding other probabilities despite the doctor's denial.

**Lessons:**

Contrast-enhanced PMCT was an effective technique with which to interpret this gastrointestinal tract rupture and served as a non-invasive tool for identifying the injury.

## 1 Introduction

Medical contrast media is conventionally used to enhance the contrast of structures within the body during medical imaging.^[1]^ In clinical practice, the application of contrast agents has become available for the central nervous system; heart and circulation; breast; lungs; gastrointestinal, genitourinary, musculoskeletal, and lymphatic systems; and even the skin.^[2]^ The use of post-mortem computed tomography (PMCT) combined with PMCT angiography (PMCTA) is also applied in forensic investigations. Contrast agents are most frequently applied to the central nervous system, heart, and circulation. However, few reports have described the application of contrast to the gastrointestinal tract. We herein report a case involving the use of contrast-enhanced PMCT for the diagnosis of intestinal perforation.

## 2 Case description

### 2.1 Case report

A 67-year-old woman with cholecystitis was hospitalized for upper abdominal cramps. Laparoscopic cholecystectomy was performed, and her abdominal cavity was explored under monitoring. Her gallbladder was then removed and packed in a specimen bag to be taken out for examination. The doctor inadvertently perforated her intestinal tract with the laparoscopic machinery. The doctor attempted to conceal the accident from the patient's family and secretly suture closed the crevasse to escape responsibility. As a result, the patient developed severe peritonitis and died about 2 weeks after the operation. An autopsy and contrast-enhanced PMCT were performed. External forensic examination and conventional autopsy were carried out. The isolated intestine was then prepared for the examinations described below.

This study was approved by the Academic Committee of the Institute of Forensic Science, Ministry of Justice, People's Republic of China. Written informed consents were obtained from the victim's family to publish these case details.

### 2.2 Contrast-enhanced PMCT examination

**Figure 1:**
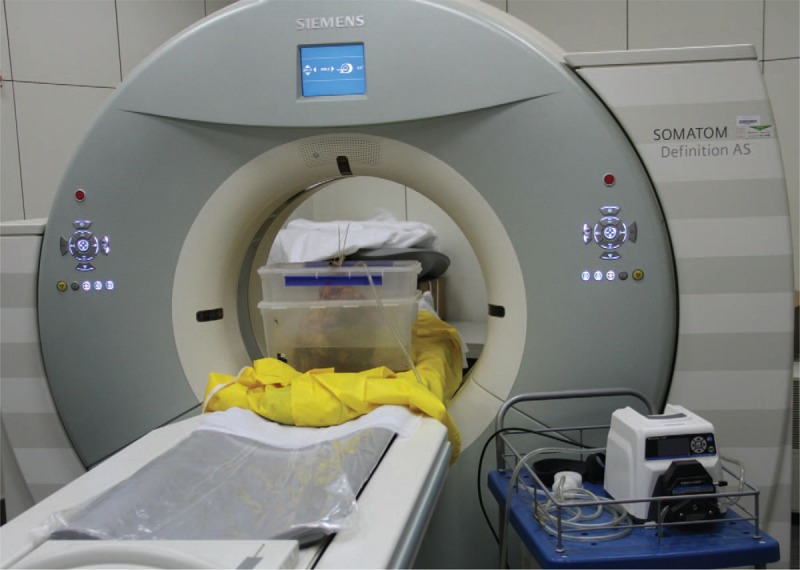
Contrast medium was injected, and the isolated intestine was supported by a handmade mounting structure. The device on the right of the figure is a special pump used to inject the contrast agent.

Contrast-enhanced PMCT of the isolated intestinal tract was carried out after the autopsy. First, we inserted a urinary catheter into the upper end and ligated the 2 ends of the intestine. The whole process was divided into 5 steps: CT scanning, air-enhanced CT scanning, water-enhanced CT scanning, formalin-unfixed intestinal CT scanning, and formalin-fixed intestinal CT scanning. The isolated intestinal tract was supported by a handmade device (Fig. 1) and scanned using a 40-slice multislice CT system (SOMATOM Definition AS; Siemens Medical Solutions, Munich, Germany). Air was then injected by an air pump, and water and contrast medium (meglumine diatrizoate and 0.9% normal saline at a 10:1 ratio) were injected through the urinary catheter. Raw data were acquired using the following settings: voltage, 120 kV; current, 240 mA; and collimation, 6.0 × 1.0 mm. Image reconstruction was achieved at slice thicknesses of 5.0 mm and 0.625 mm.

### 2.3 Autopsy findings

**Figure 2:**
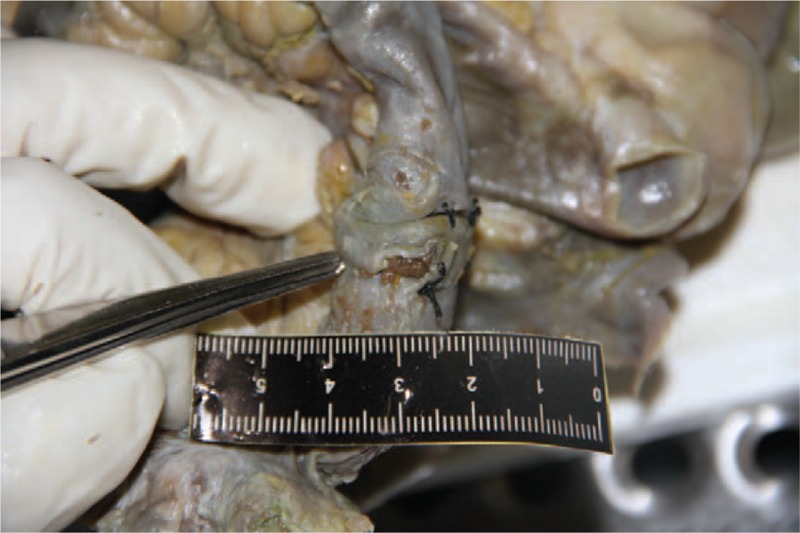
The crevasse was deep within the abdominal cavity. Squeezing the intestinal tract resulted in overflow of a small amount of intestinal contents from the crevasse.

The body was 154 cm tall and of medium build. Observations included about 400 ml of yellow turbid liquid within the abdominal cavity, omental adhesion, and a 2-cm crevasse 24 cm from the ileocecal section that was sewn with 6 stitches. No leakage of intestinal contents was detected in the crevasse. Another 0.9-cm × 0.3-cm crevasse was observed next to the stitches. The edge of the crevasse was regular, dark brown, and deep within the abdominal cavity. Squeezing the intestinal tract resulted in overflow of a small amount of intestinal contents from the crevasse (Fig. 2).

### 2.4 Radiological findings

**Figure 3:**
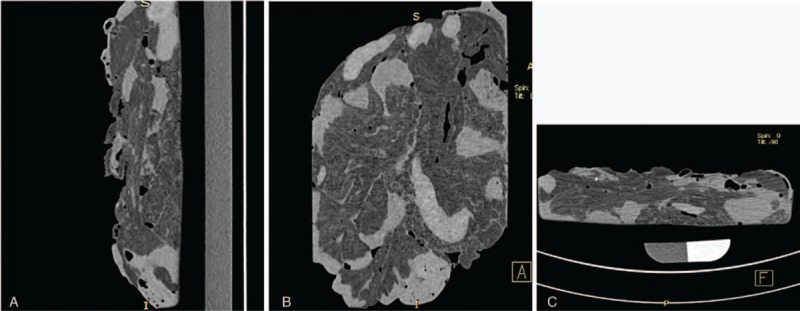
Coronal, sagittal, and cross-sectional screenshots of computed tomography images.

**Figure 4:**
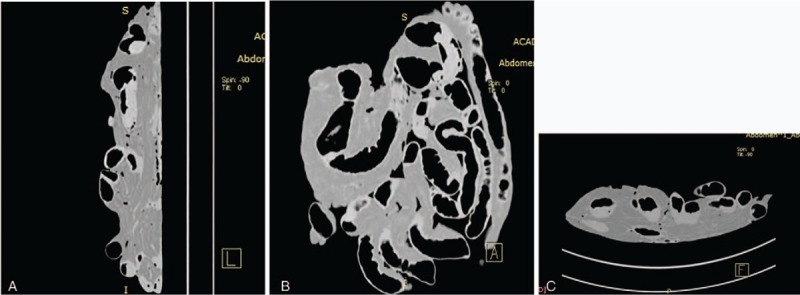
Coronal, sagittal, and cross-sectional screenshots of air-enhanced computed tomography images.

**Figure 5:**
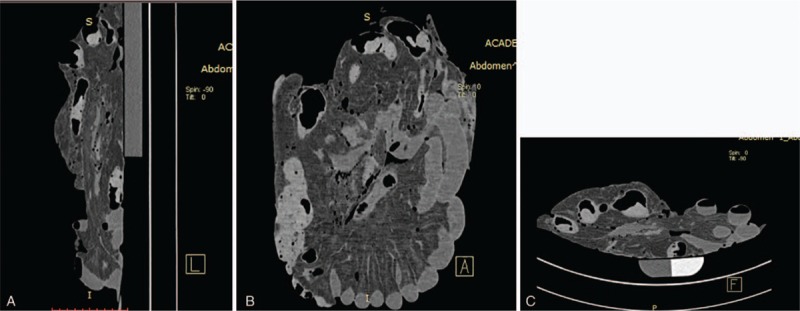
Coronal, sagittal, and cross-sectional screenshots of water-enhanced computed tomography images.

**Figure 6:**
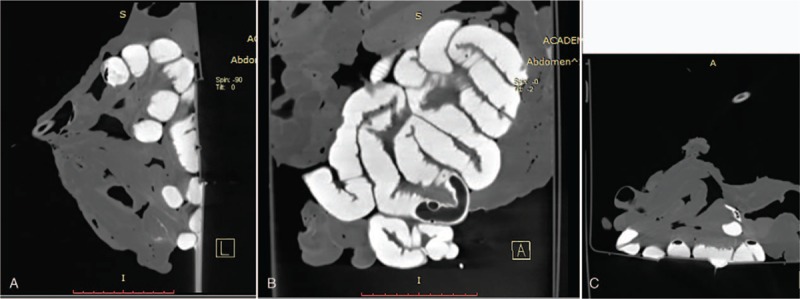
Coronal, sagittal, and cross-sectional screenshots of contrast-enhanced computed tomography images.

**Figure 7:**
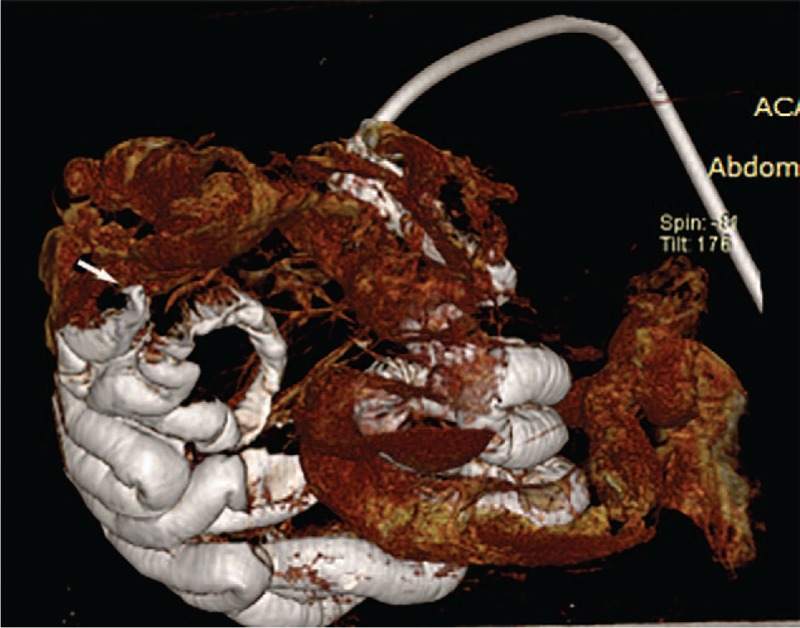
Three-dimensional volume rendering of the contrast-enhanced intestine. The white part is filled with contrast medium, and the reddish part is the contrast agent that did not enter the intestine. A trace of contrast agent flowing out of the crevasse can be detected (white arrow).

CT scanning showed partial gas-filled intestine, but most of the intestine was not obvious. No significant lesions were detected (Fig. 3). Air-enhanced CT scanning showed air overflow through the crevasse, but injected air was still present in the intestinal tract behind the crevasse because the air pump was being continuously ventilated. As a result, inflation of the entire intestinal tract increased the difficulty of finding the crevasse (Fig. 4). Water-enhanced CT scanning was then performed, and radiographs showed a gas- and water-filled intestinal tract (Fig. 5). The effect of water was not as obvious as that of contrast medium, and the result was not well appreciated. The most appreciated effect was the contrast-enhanced unfixed intestine (Fig. 6). The filling effect of formalin-fixed intestine was not as good as formalin-unfixed intestine because of the increased brittleness and expansion of the fixed intestine. In the three-dimensional volume rendering of the contrast-enhanced intestine, a trace of contrast agent flowing out of the crevasse could be detected (Fig. 7). Three-dimensional volume-rendered CT images were also obtained in the other steps.

## 3 Discussion

In the era of minimal access surgery, visceral injuries due to laparoscopic port insertion are common.^[3]^ Intestinal perforation is one such injury. The abdominal structure is anatomically complicated, and the significance of the greater omentum was recently discovered by surgeons of various disciplines, who found that it is an excellent plastic material for protection against inflammation and irradiation and for repair of abdominal cavity defects.^[4]^ However, omental adhesion of the intestine is difficult to separate in forensic practice because of its increased complexity, and whether a perforation has been caused by an accidental operation can be difficult to confirm. This may introduce subsequent complications into the forensic investigation, and the judge or doctor involved in a lawsuit may not be easily convinced by the evidence presented. Therefore, we applied contrast agent in the diagnosis of the intestinal perforation to avoid new injuries in the present case.

Contrast-enhanced imaging technology is routinely used for radiologic examinations of the gastrointestinal tract. These examinations result in rare complications because the technique is moderately invasive and not entirely innocuous.^[5]^ Virtopsy has become increasingly more widespread in forensic science and has gained a reasonable breakthrough.^[6]^ For example, PMCT and PMCTA are currently useful for elucidating injury patterns, thus providing strong medical evidence during litigation and at trial.^[7]^ However, PMCTA mostly involves the cardiovascular system; almost no reports have documented its use in the gastrointestinal tract. We, therefore, recommend applying contrast-enhanced PMCT to forensic examination of the gastrointestinal tract.

Before injecting the contrast agent, we used air and water as contrast agents because of previously proposed relevant applications using these agents. Water allows better visualization of the bowel wall than does air, which is obvious when gas is already present. Furthermore, the use of water prevents artifacts and the need for variable window settings for optimal viewing. Another advantage of the use of water is that it enables distinction between intestinal fecal material and pathologic tissue. Feces always contain small bubbles of air, and these bubbles are easily identified if the intestine is filled with liquid.^[8,9]^

The first introduction of contrast media into post-mortem investigations occurred in 2004 in the Institute of Legal Medicine in Bern, Switzerland, in the context of the virtopsy project. Investigations show that the injection of a contrast agent increases the diagnostic sensitivity of PMCT.^[10]^ In the present case, visualization of contrast-enhanced intestine was better than that using other agents. Three-dimensional volume rendering of the contrast-enhanced intestine was more illustrative, and the trace of contrast agent flowing out of the crevasse could be detected. In reality, the doctor caused lesions in 3 crevasses, but he only stitched 2 of them well. Several symptoms such as peritonitis were caused by the smallest crevasse because it was not stitched completely. In addition, the positions of the perforation and crevasse on the CT image were directly shown to the judge and doctor in court, and this image was easy to understand by even non-professionals. At first, the accused doctor doubted our conclusions. The doctor instead considered that the intestinal perforation might have been caused by enteritis or other factors related to the patient's advanced age instead of the laparoscopic cholecystectomy. However, the doctor finally agreed with us when the contrast-enhanced CT images were presented, which accurately showed the position of the perforation and the trace of the contrast media immediately next to the crevasse that was stitched. As a result, PMCT could be an effective adjunct to the postmortem radiological examination and is applicable to routine diagnosis.

## Acknowledgments

We thank Angela Morben, DVM, ELS, from Liwen Bianji, Edanz Editing China (www.liwenbianji.cn/ac), for editing the English text of a draft of this manuscript.

## Author contributions

**Funding acquisition:** Kaijun Ma.

**Methodology:** Lei Wan.

**Project administration:** Kaijun Ma.

**Resources:** Donghua Zou.

**Supervision:** Yijiu Chen.

**Writing – Original Draft:** Zhuoqun Wang.

**Writing – Review & Editing:** Ningguo Liu.
